# Differential gene expression in articular cartilage between rheumatoid arthritis and endemic Kashin–Beck disease

**DOI:** 10.1042/BSR20190188

**Published:** 2019-06-28

**Authors:** Zongqiang Gao, Chen Duan, Fang-fang Yu, Xiong Guo

**Affiliations:** 1 Orthopedic Department of The Second Affiliated Hospital of Xi’an Jiaotong University, Xi’an 710004, China; 2 Institute of Endemic Diseases, School of Public Health of Health Science Center, Xi’an Jiaotong University, Key Laboratory of Trace Elements and Endemic Diseases, National Health and Family Planning Commission, Xi’an 710061, China

**Keywords:** gene expression, immune system, Kashin-Beck disease, Rheumatoid arthritis, RNA-seq

## Abstract

Kashin–beck disease (KBD) is endemic chronic osteoarthrosis and its pathogenesis is still unclear. The present study aimed to explore differential gene expression in articular cartilage between patients with rheumatoid arthritis (RA) and KBD. Articular cartilages were collected from KBD and RA patients, and differentially expressed genes (DEGs) were analyzed by RNA-seq. The signaling pathway and biological process (BP) of the DEGs were identified by enrichment analysis. The protein–protein interaction (PPI) network of DEGs and the key genes of KBD were identified by network analysis with STRING and cytoscape software. We identified 167 immune-related DEGs in articular cartilage samples from KBD patients compared with RA. The up-regulation of MAPK signaling pathway and the down-regulation of signaling pathways such as toll-like receptor, janus kinase-signal transducers and activators of transcription, leukocyte migration, T-cell receptor and chemokine, and antigen processing and presentation were involved in KBD. We identified 137 genes nodes related with immune and mapped the PPI network diagram. BP analysis revealed that immune response, calcium ion homeostasis, blood vessel morphogenesis, inflammatory response, lymphocyte proliferation, and MAPK activation were involved in KBD. In conclusion, gene expression profiling can be used to identify the different mechanism of pathogenesis between KBD and RA.

## Introduction

Rheumatoid arthritis (RA) is a chronic inflammatory disease with an unknown cause [1,2]. The heredity, infection, or trauma may trigger an autoimmune reaction, leading to chronic inflammation in joint synovia [3–5]. A variety of immune cells such as T cells, mononuclear phagocytes and mastocytes, and several cytokines such as tumor necrosis factor α (TNF-a), interleukin (IL)-1, IL-6, IL-8, transforming growth factor β (TGF-β), and fibroblast growth factor (FGF) participate in the pathogenesis of RA [6,7]. Kashin–Beck disease (KBD) is an endemic chronic osteoarthrosis that has affected more than 640000 patients up to 2013 [8]. Previous studies found that KBD is mainly related to environmental factors such as selenium deficiency, which promote inflammatory responses [9–11]. In addition, genome-wide gene expression analysis suggested a critical role of suppressed immunity in the pathogenesis of KBD [12]. However, whole-exome sequencing showed that *HLA-DRB1* and *CD2AP* genes were implicated in KBD, indicating autoimmune response in KBD and the shared etiology between RA and KBD [13]. Furthermore, genotyping analysis revealed that *HLA-DRB1* gene variants significantly increased the susceptibility to KBD in the Tibetan population and were associated with selenium and iodine deficiencies [14].

To further understand the similarity and difference of molecular mechanisms between KBD and RA, in the present study we performed RNA-seq analysis to compare the differentially expressed genes (DEGs) in the articular cartilages from KBD and RA patients. Our results provide new evidence for the differences in immune function between KBD and RA patients and shed new insight into the pathogenesis of KBD and RA.

## Materials and methods

### Subjects

The present studywas approved by the Human Ethics Committee of Xi’an Jiao Tong University, and all patients signed informed consent. RA patients were from the non-KBD-endemic areas in Xi’an, while KBD patients were from KBD-endemic areas of Linyou and Yongshou in Shaanxi province of Northwest China. The revised diagnosis criteria (Rheumatoid Arthritis Classification Criteria 2010) were used for the identification of patients with RA [15]. KBD patients originated from the endemic areas based on the diagnosis criteria of KBD (WS/T207-2010) without other arthritic diseases. KBD and RA samples were collected from the discarded cartilage tissue during total knee replacement in the hospital from eight pairs of KBD and RA patients, all Chinese Han lineage. Samples from three pairs of patients (KBD patients of 56-year old female, 57-year old female, and 61-year old male, matched to RA patients of 56-year old female, 57-year old female, and 62-year old male) were used for RNA-seq analysis, and samples from additional five pairs of KBD and RA patients with matched gender and age (three males and two females, average age 56.8 vs 60.2 years) were used for subsequent confirmation by qRT-PCR analysis.

### RNA-seq analysis

Cartilage specimens were pulverized into powder in liquid nitrogen, and total RNA was extracted and purified using TRIzol kit (Invitrogen, Carlsbad, CA, U.S.A.) according to the manufacturer’s protocol. A library was established for each sample and sequenced using Illumina Nextseq 500 RNA Sample Preparation Kit (Illumina, San Diego, CA, U.S.A.) according to the manufacturer’s instructions.

### Real-time qRT-PCR

Total RNA was isolated as described previously [16]. cDNA was synthesized using Superscript II reverse transcriptase (Invitrogen, Carlsbad, CA, U.S.A.) and random primers. ABI 7500 RT- PCR System (Applied Biosystems, Foster City, CA, U.S.A.) was used for real-time qRT-PCR analysis and glyceraldehyde-3-phosphate dehydrogenase (GAPDH) as an endogenous control.

### Gene-disease associations and signaling pathways

The Database for Annotation, Visualization, and Integrated Discovery (DAVID) (https://david.ncifcrf.gov/home.jsp) provides an integrated and expanded back-end annotation database, advanced modular enrichment algorithms, and powerful exploratory ability in an integrated data-mining environment. Therefore, DAVID was utilized for the gene-disease (classification) association analysis based on the identified DEGs of KBD with κ > 0.75. In addition, DAVID was used for the enrichment of KEGG signaling pathway.

### Protein–protein interaction

The STRING database (http://string-db.org) are a resource for the assessment and integration of protein–protein interaction (PPI), including both direct (physical) and indirect (functional) interactions in an organism. First, the DEGs were input into STRING database to construct PPI network. Next, PPI network was reconstructed using Cytoscape software version 3.3.0 (http://www.cytoscape.org/). The connectivity degree of each protein node in PPI network was calculated and the top hub nodes were identified using the Cytoscape plugin Network Analyzer (http://www.cytoscape.org/).

## Results

### Differential gene expression between KBD and RA

**Figure 1:**
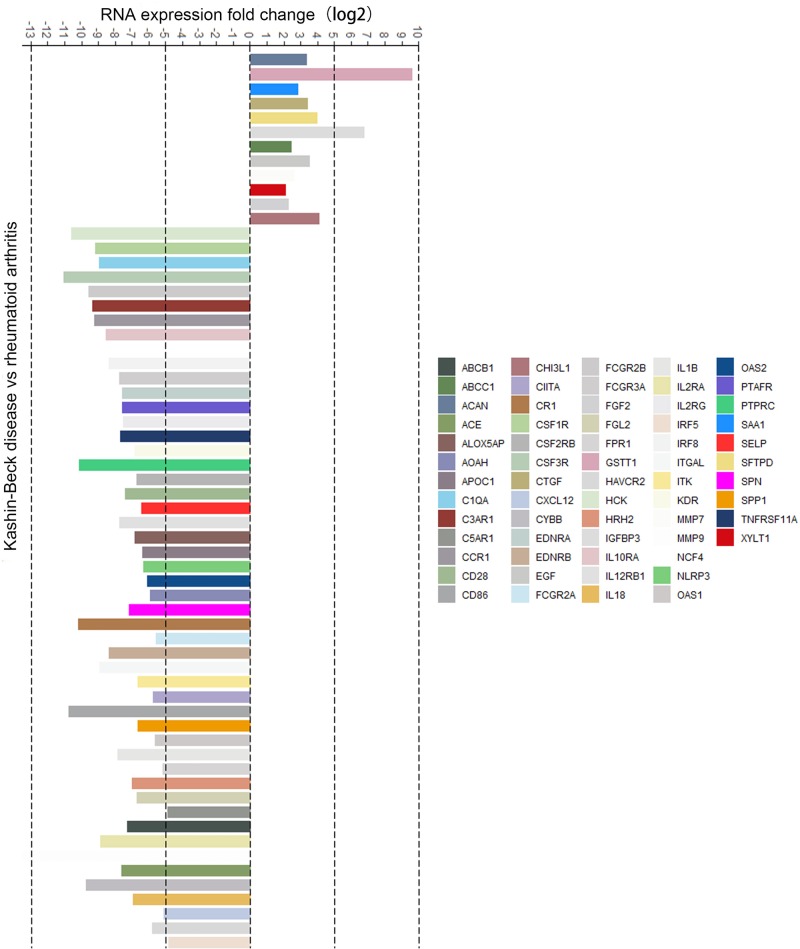
The selected list of significantly up-regulated and down-regulated immune-related genes in KBD cartilage Most of the genes were down-regulated in KBD cartilage compared with RA cartilage, suggesting that immune function suppression contributes to the pathogenesis of KBD.

To explore differential gene expression in articular cartilage from KBD patients compared with RA patients, we performed RNA-seq analysis and identified 232 up-regulated and 1335 down-regulated genes in KBD compared with RA. DAVID analysis of these 1567 DEGs in KBD divided them into four categories: IMMUNE (167 genes), INFECTION (79 genes), CARDIOVASCULAR (122 genes), and HEMATOLOGICAL (31 genes) (*P*<0.05) (Table 1). For the category, we considered the κ > 0.75 as significant. Therefore, the category of IMMUNE in KBD with 167 DEGs was selected (Figure 1).

**Table 1:** Genetic (class) association with KBD

Category	Gene (%)	*P*-value^*^	*P*-value^†^	κ
IMMUNE	167 (11.1)	7.2 × 10^−13^	1.3 × 10^−11^	1.00
INFECTION	79 (5.2)	2.1 × 10^−10^	1.9 × 10^−9^	0.46
CARDIOVASCULAR	122 (8.1)	5.4 × 10^−5^	3.3 × 10^−4^	0.39
HEMATOLOGICAL	31 (2.1)	5.7 × 10^−3^	2.5 × 10^−2^	0.38

* Nominal *P*-value was calculated by hypergeometric test.

† Adjusted *P*-values was corrected of nominal *P*-values by Benjamini–Hochberg multiple testing correction.

### Function enrichment analysis

To understand the role of the identified DEGs in the pathogenesis of KBD, we performed KEGG signaling pathway enrichment analysis. For the 167 immune-related DEGs between KBD and RA, we identified one up-regulated MAPK signaling pathway involving 15 up-regulated genes, and 7 down-regulated signaling pathways involving 152 down-regulated genes (*P*<0.05), which included toll-like receptor (TLR) signaling pathway, janus kinase-signal transducers and activators of transcription (JAK-STAT) signaling pathway, leukocyte transendothelial migration, NOD-like receptor signaling pathway, T-cell receptor signaling pathway, chemokine signaling pathway, and antigen processing and presentation (Table 2).

**Table 2:** KEGG signaling pathways significantly enriched in KBD compared with RA

KEEG pathway	Number of genes (%)	*P*-value^*^
Up-regulation		
MAPK signaling pathway	3 (20)	4.8 × 10^−2^
Down-regulation		
TLR signaling pathway	12 (7.9)	2.3 × 10^−5^
Jak-STAT signaling pathway	14 (9.2)	6.7 × 10^−5^
Leukocyte transendothelial migration	11 (7.2)	4.5 × 10^−4^
NOD-like receptor signaling pathway	8 (5.3)	6.0 × 10^−4^
T-cell receptor signaling pathway	10 (6.6)	9.8 × 10^−4^
Chemokine signaling pathway	13 (8.6)	1.5 × 10^−3^

* Nominal *P*-value was calculated by hypergeometric test.

### PPI network

**Figure 2:**
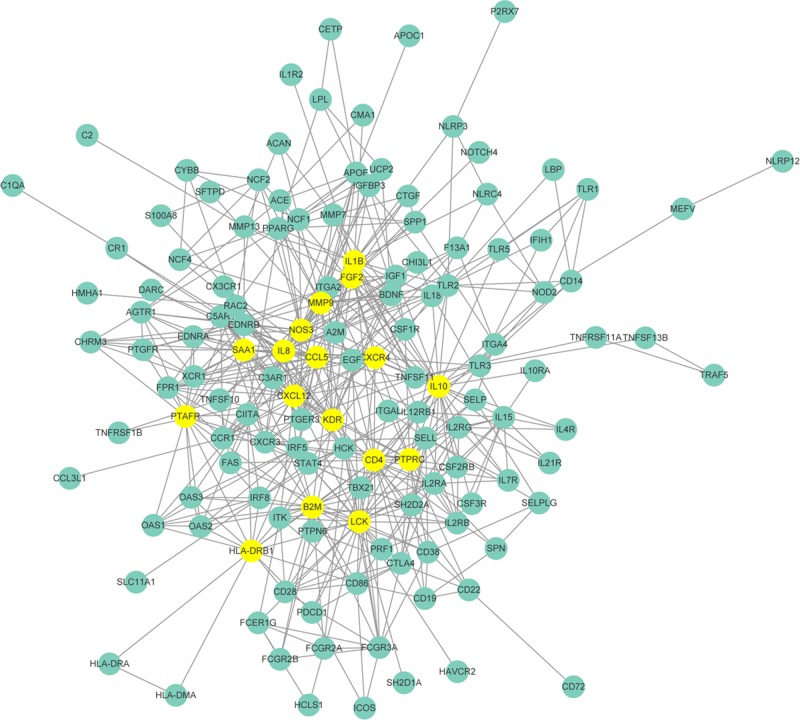
PPI network of 167 DEGs STRING PPI network analysis of 167 DEGs in KBD led to the identification of 137 nodes (presented as a circle). The top 17 nodes (hub nodes) with wide range of interactions were marked in yellow and also listed in Table 3.

To reveal functional interaction of the identified DEGs, we performed PPI network analysis and identified 137 gene nodes in KBD for the 167 DGEs, forming a complex structure of multicenter interaction network of DEGs. Notably, 17 DEGs (marked as yellow) were identified as hub nodes as they not only interacted with the surrounding nodes in the network, but also had a wide range of interactions with other hub nodes (Figure 2). As shown in Table 3, these 17 DEGs (hub nodes) were widely involved in various biological processes.

**Table 3:** The 17 key immune-related genes were identified in KBD cartilage

Gene	Gene symbol	Log_2_ fold-change	*P*-value^*^
Glutathione S-transferase θ 1	SAA1	2.82	4.30 × 10^−3^
Fibroblast growth factor 2	FGF2	2.24	4.48 × 10^−2^
β-2-microglobulin	B2M	−3.31	6.78 × 10^−3^
Chemokine (C–X–C motif) receptor 4	CXCR4	−3.81	1.64 × 10^−4^
CD4 molecule	CD4	−4.33	8.73 × 10^−4^
Interleukin 10	IL10	−4.61	7.29 × 10^−5^
chemokine (C–X–C motif) ligand 12	CXCL12	−5.20	1.53 × 10^−5^
kinase insert domain receptor	KDR	−6.90	7.98 × 10^−9^
lymphocyte-specific protein tyrosine kinase	LCK	−7.05	6.39 × 10^−5^
interleukin 8	IL8	−7.10	1.03 × 10^−2^
nitric oxide synthase 3	NOS3	−7.39	1.13 × 10^−3^
chemokine (C-C motif) ligand 5	CCL5	−7.40	2.12 × 10^−2^
platelet-activating factor receptor	PTAFR	−7.66	1.86 × 10^−9^
interleukin 1, β	IL1B	−7.94	1.86 × 10^−9^
protein tyrosine phosphatase, receptor type, C	PTPRC	−10.18	1.03 × 10^−8^
matrix metallopeptidase 9	MMP9	−13.54	5.52 × 10^−6^
major histocompatibility complex, class II, DR beta1	HLA-DRB1	−13.63	1.50 × 10^−2^

* Nominal *P*-value was calculated by hypergeometric test.

### Confirmation of the expression of ten DEGs identified in KBD

**Figure 3:**
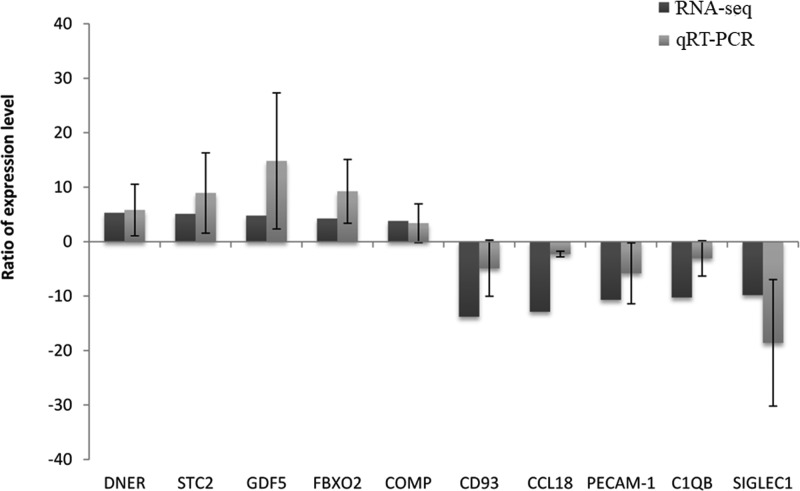
Confirmation of representative DEGs by qRT-PCT analysis The five genes up-regulated in KBD (left five columns) and five genes down-regulated in KBD (right five columns) identified by RNA-seq analysis were selected and their expression levels were detected by qRT-PCR analysis. The up-regulation and down-regulation of these genes in KBD cartilage compared with RA cartilage showed consistency as detected by either RNA-seq analysis or qRT-PCR analysis.

To confirm that RNA-seq is a powerful approach to identify DEGs, we randomly selected ten DEGs identified by RNA-seq analysis. By qRT-PCR analysis, we found that five genes (*DNER, STC2, GDF5, FBXO2*, and *COMP*) identified to be up-regulated from cartilage tissue of KBD patients by RNA-seq analysis showed the change of up-regulation, while five genes (*CD93, CCL18, PECAM-1, C1QB*, and *SIGLEC1*) identified to be down-regulated from cartilage tissue of KBD patients by RNA-seq analysis showed the change of down-regulation (Figure 3). These data demonstrate that the results of RNA-seq analysis are consistent with qRT-PCR results.

## Discussion

Gene expression profiling has been increasingly used to reveal molecular mechanism underlying physiological and pathological processes [16–18]. In the present study, we employed RNA-seq analysis to screen the DEGs in KBD compared with RA. In addition, the expression of ten randomly selected genes was validated by qRT-PCR analysis. The KEGG signaling pathway enrichment analysis revealed that the key up-regulated genes (*SAA1* and *FGF2*) were involved in the MAPK signaling pathway, and down-regulated genes (*HLA-DRB1, MMP9, PTPRC, IL1B, PTAFR, CCL5, NOS3, IL8, LCK, KDR, CXCL12, IL10, CD4, CXCR4*, and *B2M*) were involved in JAK-STAT-, TLR-, T-cell receptor, and chemokine signaling pathways.

MAPK signaling pathway can be activated by extracellular signal or physical stimuli such as stress, inflammatory cytokines and growth factors to promote cell proliferation, differentiation, and migration. In human, there are four distinct groups of MAPKs, including the extracellular signal-related kinases (ERK)-1/2, Jun amino-terminal kinases (JNK1/2/3), p38 proteins (p38), and ERK5 [19]. The JNK and p38 play critical role in the stimulation of inflammatory response. The higher mRNA levels of p38 and JNK observed in the present study were consistent with previous findings that the levels of p-p38 and p-JNK increased in KBD cartilage compared with healthy individuals [20]. During cartilage injury, oxidative stress and inflammatory cytokine can stimulate JNK and p38 MAPK signaling pathways, resulting in chondrocyte apoptosis. In addition, FGF 2 (FGF2) in the extracellular matrix of chondrocyte and osteoblast plays an important role in chondrocyte differentiation, cartilage matrix synthesis, and the coordination of osteoblast and osteoclast differentiation and function [21,22]. Immunohistochemical analysis showed that bFGF expression was enhanced in the middle and deep zones of articular cartilage in children with KBD and further expanded to the upper zone in KBD adults compared with normal cartilage [23]. In addition, microarray analysis showed that FGF2 was significantly up-regulated in the cartilages of KBD patients compared with healthy controls [24]. Therefore, the activation of MAPK pathway by FGF2 may promote chondrocyte apoptosis.

Interestingly, the 167 immune-related genes included 15 up-regulated genes and 152 down-regulated genes in KBD compared with RA. These data suggest that immune function suppression plays a critical role in the pathogenesis of KBD, consistent with previous results [12]. The down-regulated pathways included JAK-STAT, TLR, T-cell receptor, and chemokine signaling pathways. Notably, the expression of IL-6, IL-15, and granulocyte macrophage colony stimulating factor receptor (GM-CSF) in the synovial of RA patients was significantly up-regulated by JAK-STAT pathway, which contributed to joint destruction in RA pathogenesis [25]. In addition, the activation of STAT pathway exhibited anti-apoptosis effects [26]. Our results suggest that JAK-STAT pathway plays opposite role in KBD and RA, and the down-regulation of STAT pathway may promote chondrocyte apoptosis.

For other down-regulated pathways, the chemokines are known to regulate inflammatory cell migration and activate fibroblast-like synoviocytes in RA synovium [27]. TLR is a crucial part of innate immune system by driving efficient T-cell response to the pathogens. Many downstream components of TLR are the same as those of T-cell receptor, and these two pathways function in concert to activate potent T-cell response [28]. The immune damage caused by the activation of T-cell receptor plays an important role in RA [29]. In contrast, the role of chemokine, TLR, and T-cell receptor in KBD remains elusive. Our findings of the down-regulation of these pathways in KBD suggest that further studies on these pathways are worthy to elucidate the immune dysfunction involved in KBD.

In summary, gene expression profiling can be used to identify the different pathogenesis between KBD and RA. The DEGs and pathways we identified will be candidate targets for effective therapy of KBD.

## Abbreviations

BP: biological process
DEG: differentially expressed gene
ERK: extracellular signal-related kinase
FGF: fibroblast growth factor
IL: interleukin (IL)-1
JAK-STAT: janus kinase-signal transducers and activators of transcription
JNK: Jun amino-terminal kinase
KBD: Kashin–beck disease
PPI: protein–protein interaction
RA: rheumatoid arthritis
